# Effective Piecewise Mass Distributions for Optimal Energy Eigenvalues of a Particle in Low-Dimensional Heterojunctions

**DOI:** 10.3390/nano14221850

**Published:** 2024-11-20

**Authors:** Josep Batle, Orion Ciftja, Mahmoud Abdel-Aty, Mohamed Ahmed Hafez, Shawkat Alkhazaleh

**Affiliations:** 1 Departament de Física and Institut d’Aplicacions Computacionals de Codi Comunitari (IAC3), University of Balearic Islands, E-07122 Palma de Mallorca, Spain; jbv276@uib.es; 2 CRISP—Centre de Recerca Independent de Sa Pobla, E-07420 Mallorca, Spain; 3 Department of Physics, Prairie View A&M University, Prairie View, TX 77446, USA; 4 Department of Mathematics, Faculty of Science, Sohag University, Sohag 82524, Egypt; mabdelaty@zewailcity.edu.eg; 5 Deanship of Graduate Studies and Research, Ahlia University, Manama 10878, Bahrain; 6 Department of Civil Engineering, Faculty of Engineering, INTI International University, Nilai 71800, Malaysia; mohdahmed.hafez@newinti.edu.my; 7 Department of Mathematics, Faculty of Science and Information Technology, Jadara University, Irbid 21110, Jordan; s.alkhazaleh@jadara.edu.jo

**Keywords:** nanomaterials, heterojunction, effective mass, position-dependent mass, quantum well

## Abstract

Systems composed of several multi-layer compounds have been extremely useful in tailoring different quantum physical properties of nanomaterials. This is very much true when it comes to semiconductor materials and, in particular, to heterostructures and heterojunctions. The formalism of a position-dependent effective mass has proved to be a very efficient tool in those cases where quantum wells emerge either in one or two dimensions. In this work, we use a variety of mathematical theorems, as well as numerical computations, to study different scenarios pertaining to choices of a specific piecewise constant effective mass for a particle that causes its energy eigenvalues to reach an extremum. These results are relevant when it comes to practical technological applications such as modifying the optical energy gap between the first excited state and the ground state energy of the system. At the end of our contribution, we also question the physical validity of some approximations for systems with particles that possess a position-dependent mass especially for those cases in which the mass distribution is divergent.

## 1. Introduction

The concept of effective mass for electrons in a semiconductor material is familiar to many of those working in physical sciences and/or engineering disciplines. In solid-state physics, the effective mass is a quantity that is used to simplify band structures by constructing an analogy to the behavior of a free particle. In the simplest of the cases, the effective mass of an electron in a semiconductor may be constant and isotropic or constant and anisotropic [1,2]. Sometimes, the effective mass of the electron in a host semiconductor is very different from its bare mass though all depends on the specific nature of the semiconducting materials under consideration. For instance, electrons in some III–V compounds such as GaAs and InSb have far smaller effective masses than their counterparts in tetrahedral group IV materials like Si and Ge. This is relevant because the ultimate speed of integrated circuits depends on the carrier velocity, which is inversely proportional to the effective mass. Therefore, the low effective mass of electrons in III–V compounds explains why GaAs and its derivatives are used in high-bandwidth applications instead of Si.

On the other hand, the study of quantum systems consisting of electrons with position-dependent effective masses is much more difficult and has received considerable attention in recent years [3,4,5]. In this work, we consider a quantum particle such as an electron that possesses a position-dependent mass. We study specific situations that arise when solving the resulting quantum problem in a one-dimensional (1D) or a two-dimensional (2D) space. For the 1D case, the position-dependent mass of the electrons is different in different 1D regions. The simplest choice considered is a piecewise constant function for the mass meaning that the function has different values in different regions but is locally constant within any given region. We assume circular symmetry for the corresponding 2D case scenario in order to keep the problem analytically treatable up to a certain point. Although most of the ideas concerning the position-dependent mass concept were conceived in the domain of solid-state physics [6,7,8,9,10,11], the approach has naturally spread to other subjects [12,13,14,15,16]. The resulting stationary Schrödinger’s equation for a particle with a non-constant mass provides an interesting and useful model for the description of many physical problems especially in the case of semiconductor nanostructures. One of the most popular of these structures is the semiconductor quantum well where Schrödinger’s equation there is effectively 1D. The 1D finite well potential [17] can be modeled in such a way, as the wave function is approximately zero at the boundaries. Nevertheless, one must be cautious about this idealization and remark that the approach should not be treated very strictly because otherwise, there would not be any transport and/or tunneling phenomena in such structures.

The amount of studies of quantum systems containing particles with position-dependent masses has greatly increased in the last decade due to the widening of the range of potential technological applications [18,19,20,21,22,23]. Therefore, it is safe to say that there are several areas where intensive work is being conducted. Among them, we may mention efforts to understand the electronic properties of semiconductor heterostructures, quantum wells and quantum dots [24,25,26,27,28,29,30,31,32,33,34,35,36,37,38,39,40,41], crystal-growth [42], helium clusters [43], quantum liquids [44,45], as well as research in superconductors [46,47,48,49,50,51]. In particular, low-dimensional carrier systems in semiconductor heterostructures have gained great importance due to the potential use of their unique properties in applications ranging from optoelectronics to highspeed devices [52,53,54]. For example, InGaN/GaN quantum well structures are of great interest to various technologies because their band gap can be tailored to cover a wide spectral range from red to ultraviolet by changing the composition of In.

The objective of the present work is to determine pertinent features that apply to a quantum particle with position-dependent mass confined in a 1D or 2D structure with the understanding that such features lead to an optimal ground state and first excited energy eigenvalue. We consider a particle with mass that depends on the position coordinate for the 1D case and a position-dependent mass with radial symmetry for the 2D case scenario. This allows us to use a functional form similar to that in 1D when considering a position-dependent mass in 2D with the assumption that the mass is a function of the radial coordinate. In this study, we consider a particle with position-dependent mass that is a piecewise constant. Our analysis of the results includes various scenarios for such a mass distribution. The external confinement potential that we will consider is constant or piecewise constant. For simplicity, we focus the attention on a single region and assume that the potential there is zero while infinite walls are applied at the boundaries. Therefore, the focus of the current study is the mere form of the position-dependent mass.

This article is structured as follows: Section 2 deals with the mathematical description of the ensuing Schrödinger’s equation so that the problem is well-posed. Section 3 describes some specific position-dependent mass models that are used in this study. Results for 1D structures are presented in Section 4. Corresponding results for 2D structures are shown in Section 5. Finally, some brief discussions and conclusions are drawn in Section 6.

## 2. Mathematical Statement of the Problem

The 1D stationary Schrödinger’s equation for a particle with constant mass, for instance, an electron with bare mass, $m_{e}$ reads

(1) $$\frac{{\hat{p}}_{z}^{2}}{2\;m_{e}}\;\psi \left(z\right)+V\left(z\right)\;\psi \left(z\right)=E\;\psi \left(z\right)\;,$$

where ${\hat{p}}_{z}$ is the linear momentum operator along the *z* direction, V(z) is an external potential, *E* is the energy eigenvalue, and ψ(z) is the wave function. Extension of Equation (1) to the case of a quantum particle with position-dependent mass, m(z) is not a straightforward process since the linear momentum operator, ${\hat{p}}_{z}$ in general, does not commute with m(z). Quantum mechanical rules require the kinetic energy operator to be Hermitian. A very general form of this operator that satisfies such a condition is

(2) $$\hat{T}=\frac{1}{4}\left[m^{a}\left(z\right)\;{\hat{p}}_{z}\;m^{b}\left(z\right)\;{\hat{p}}_{z}\;m^{c}\left(z\right)+m^{c}\left(z\right)\;{\hat{p}}_{z}\;m^{b}\left(z\right)\;{\hat{p}}_{z}\;m^{a}\left(z\right)\right]\;,$$

where a+b+c=−1. The simplest form of the kinetic energy operator is obtained when a=c=0 and b=−1, leading to the following expression:

(3) $$\hat{T}=\frac{1}{2}\left[{\hat{p}}_{z}\frac{1}{m(z)}{\hat{p}}_{z}\right]\;.$$

The choice in Equation (3) is the most common selection for particles with position-dependent effective masses in semiconductor theory [55]. Any other more general forms lead to more challenging differential equations to handle. More extensive discussions regarding the various forms of the kinetic energy operator and the ordering ambiguity can be found in Refs. [56,57].

In particular, the above two works [56,57] discuss at length the problem of a 1D classical and quantum harmonic oscillator for a particle with position-dependent mass. For example, Ref. [57] studies the problem of a classical and quantum 1D harmonic oscillator for a particle with position-dependent mass by using a super-symmetric approach that involves properly constructed operators. The correspondence between the classical and the quantum Hamiltonians is used to fix the ordering of the kinetic energy terms in the quantum framework. Examples of specific position-dependent mass functions with no singularities and with singularities are also studied.

If the particle is free but confined by infinite walls in the 0≤z≤L region, the potential in that region may be written as

(4) V(z)=0;0≤z≤L.

As a result, stationary Schrödinger’s equation describing a particle with position-dependent effective mass in a 1D infinite quantum well becomes

(5) $$-\frac{\hbar^{2}}{2}\nabla \left[\frac{1}{m(z)}\nabla \right]\psi \left(z\right)=E\;\psi \left(z\right)\;,$$

where the nabla operator in 1D is ∇=∂/∂z.

For simplicity, we assume that atomic units are used. This way, the energy is measured in units of a Hartree ($k\;e^{2}/a_{B}$), where *k* is Coulomb’s electric constant, *e* is the electron’s charge, and $a_{B}$ is the Bohr radius. The distances are measured in units of $a_{B}$. Use of atomic units means that one can formally set $\hbar =m_{e}=e=k=1$ and, as a result, the notation for Equation (5) further simplifies.

The above problem can be written in a more general mathematical form as

(6) $$\begin{array}{c} -\nabla \left[\frac{1}{m(z)}\nabla \psi \left(z\right)\right]=\lambda \left(m(z)\right)\psi \left(z\right)\;\;\mathrm{in}\;\Omega \;,\\ \psi (z)=0\;\mathrm{on}\;\partial \Omega \;, \end{array}$$

where Ω is a bounded domain in $R^{N}$, N≥1, ∂Ω is the boundary of that domain, and m(z) is a positive function that represents the effective position-dependent mass of the particle. Notice that the eigenvalue λ is explicitly written as a functional of the input mass function, m(z) for clarity. We employ the usual notation used in mathematics for elliptic differential equations to adhere to the theorems that we shall apply. Notice that any other form for the position-dependent mass that is different from that of Equation (6) renders the mapping to elliptic theory of little use. The boundary condition considered in Equation (6) is that of the Dirichlet (absorbing) type. This would be consistent with the continuity condition of the wave function with the understanding that the particle is confined by infinite walls. Quantum rules make this choice for the wave function mandatory but do not impose any condition on the derivative of the wave function. This means that a Neuman-type reflecting boundary condition for the adequate derivative must be excluded for such a case. An interesting discussion with regard to these two classes of boundary conditions from the perspective of a classical statistical case involving a fractal-like and finite system is found in Ref. [58].

This mathematical problem has countably many real positive eigenvalues with finite multiplicity which diverge to infinity. These eigenvalues are explicitly computable only for a handful of domains (such as a disk or a rectangle) when m(z) is constant or piecewise constant. Therefore, this difficulty motivated the research to look for a function m(z) within a given class of functions which can minimize or maximize (leading to an extremum) the eigenvalues of the problem in Equation (6). The aim of the present work is to look for specific values of the piecewise constant mass of the electron that can maximize the ground state energy of the electron for the given constraints that are imposed in the system. The maximization of the ground state energy of an electron with piecewise constant mass for the 1D system under consideration is a direct result of the application of the variational principle in the theory of elliptic equations. In the same vein, and strictly within physical applications, one could also opt to minimize the ground-state energy. This would imply appealing to a lower energy value and, perhaps, a more stable system. A much more interesting case is that of engineering the difference, $\Delta \equiv E_{1}-E_{0}$ between the first pair of neighboring energy levels which is directly linked to tunneling effects. But this instance is out of the scope of the present approach for it involves two energy levels and it departs from the mathematical theory that supports a single eigenvalue. An important tool in this direction is given by the following classical minimum-maximum characterization, as follows:

(7) $$E_{0}\left(m\right)=\min_{\begin{array}{c} y\neq 0\\ y\in H_{0}^{1}\left(\Omega \right) \end{array}}\frac{\int_{\Omega }\frac{1}{m(z)}{\left|\nabla y(z)\right|}^{2}dz}{2\int_{\Omega }y{\left(z\right)}^{2}dz}$$

and

(8) $$E_{k}\left(m\right)=\min_{\begin{array}{c} V_{k+1}\subset H_{0}^{1}\left(\Omega \right)\\ \dim V_{k+1}=k+1 \end{array}}\max_{\begin{array}{c} y\neq 0\\ y\in V_{k+1} \end{array}}\frac{\int_{\Omega }\frac{1}{m(z)}{\left|\nabla y(z)\right|}^{2}dz}{2\int_{\Omega }y{\left(z\right)}^{2}dz}\;.$$

Since the main goal of the current work is an optimization study, we will employ the above mathematical tools, which are borrowed from the theory of variational formulation of elliptic partial differential equations. Details about formalism and rationale behind these equations can be found in Ref. [59] and references therein. At this juncture, we remark that the function $m⟶E_{k}\left(m\right)$ is decreasing, namely, $m_{1}\leq m_{2}$ implies $E_{k}\left(m_{1}\right)\geq E_{k}\left(m_{2}\right)$. Therefore, there is no interest to study the minimization or maximization problem for $E_{k}\left(m\right)$ in a class of type, as follows:

(9) $$F=\left\{m\in L^{\infty }\left(\Omega \right):\alpha \leq m\left(z\right)\leq \beta \;a.e.\;\mathrm{in}\;\Omega \right\}\;,$$

since the obvious solution will be m(z)=β or m(z)=α. Therefore, in this paper, we will be dealing with optimization problems for the eigenvalues of Equation (6) in the following class:

(10) $$F_{\alpha ,\beta ,c}=\left\{\begin{array}{c} m\in L^{\infty }\left(\Omega \right):0<\alpha \leq m\left(z\right)\leq \beta \;a.e.\;\mathrm{in}\;\Omega \;,\\ \int_{\Omega }m\left(z\right)\;dz=c \end{array}\right\}\;,$$

where the constant *c* will be given and “a.e.” means “almost everywhere ”.

## 3. Mass Distribution Modeling

The problem of having a position-dependent mass in stationary Schrödinger’s equation with the Dirichlet boundary conditions implies that we must impose the continuity of the wave function between regions. In addition, one must consider special matching conditions between the slopes and masses on either side. For instance, when a heterojunction is formed by two different semiconductors, a quantum well can be fabricated at the interface due to the difference in the band structure. In order to calculate the energy levels within the quantum well, it is of great importance to understand the mismatch of the effective mass, $m^{*}$ across the junction. The reported boundary condition for the envelope function in a quantum well is known as the BenDaniel–Duke boundary condition [60]. The envelope function for such a boundary condition must be such that ψ(z) and $\frac{1}{m^{*}}\frac{\partial }{\partial z}\psi \left(z\right)$ must be continuous on both sides of the interface region.

One way to avoid the numerical burden associated with these boundary conditions is to have a smooth, continuous, and differentiable mass distribution everywhere. In those cases where we shall deal with step-wise functions, we employ instead a form of the type $\tanh[k\;\left(z-z_{0}\right)]$ where the parameter, *k* (k=5000 in our case) is large enough so that the differences between the obtained eigenvalues and the ones using the matching conditions are absolutely negligible. Needless to say, we can also assume a particular form of the position-dependent mass, m(z) such that it is a continuous and monotonic function with results that can even be obtained analytically.

**Figure 1:**
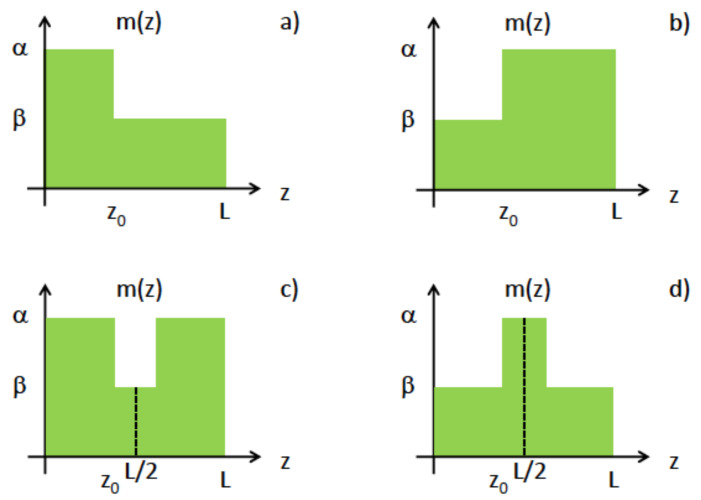
(Color online.) Different step-like mass distributions proposed for either 1D or 2D systems. (**a**–**d**) These are the four simplest stepwise functions used in this work. See text for details.

Since we will be dealing with a 1D or a 2D problem with radial symmetry, the four simplest step-wise functions used in each case are those depicted in Figure 1. The 2D quantum problem reduces to a 1D mathematical problem if the position-dependent mass of the particle depends only on the radial coordinate, but not the polar angle. Thus, one employs the radial symmetry of the mass to reduce the problem to a 1D scenario that involves the 2D radial direction only. An interesting discussion of how symmetry leads to a dimensional reduction of this type can be found in Ref. [61].

Since the objective of the work is to compare eigenvalues with the case of constant mass, $m_{0}=1$, we choose L=2 so that all areas in Figure 1 are equal to $2\;m_{0}=2$. The choice of L=2 is motivated by the fact that results will be symmetric with respect to the mid-point.

We use standard Numerov and Runge–Kutta methods [62] to obtain the numerical solution of the resulting stationary Schrödinger’s differential equations. This means that we have double-checked the numerical accuracy of the results that we obtained. These two numerical methods represent robust tools to solve eigenvalue-eigenfunction problems of the nature encountered in our work. Both methods require truncating and discretizing a region of space that is normally spanned by an infinite dimensional Hilbert space. The Numerov method is a finite difference method that calculates the shape of the state by integrating step-by-step along a grid. The Runge–Kutta method is an alternative popular choice which has the ability to increase the accuracy of the calculations at the expense of more iterations. We employed the fourth-order variant of the Runge–Kutta method to achieve the utmost accuracy. The configurations studied in Figure 1 are the simplest non-trivial instances for piecewise constant mass distribution functions for which the maximum principle is applied in the theory of elliptic differential equations. Other instances would render this study unnecessarily complex and would blur the mapping between mathematical theory and piecewise mass design.

The required parameters are β/α and $z_{0}$. This means that α and β are uniquely defined in terms of β/α and $z_{0}$ (because all areas are the same). Special attention must be paid to the symmetric cases for $z_{0}$ ranges that are between 0 and 1, not between 0 and 2. The parametrization employed is the following: (i) single jump, $\alpha =2/(\left(2-z_{0}\right)\beta /\alpha +z_{0})$ and $\beta =2\beta /\alpha /(\left(2-z_{0}\right)\beta /\alpha +z_{0})$; (ii) double jump, $\alpha =1/(\left(1-z_{0}\right)\beta /\alpha +z_{0})$ and $\beta =\beta /\alpha /(\left(1-z_{0}\right)\beta /\alpha +z_{0})$. These values ensure that the area under the mass curves is equal to 2. This corresponds to a constant mass of $m_{0}=1$ up to L=2 either in 1D or in the 2D case of a mass with radial symmetry that we will study later on.

Armed with these tools, we are now in a position to solve the problem of a particle with position-dependent mass either in 1D or 2D with the aim of obtaining those distributions that optimize the first eigenvalue, $E_{0}$, as well as the next eigenvalue in terms of the first one; that is, $E_{1}/E_{0}$. These quantities can be relevant when designing a semiconductor device for a specific use. This is very much the case when the mass dependency on position comes in the form of a stepwise function, which can be achieved experimentally.

One very important point here is to acknowledge that our discussion for the 2D case relies on a kinetic energy term of the form $-\frac{1}{2}\nabla \frac{1}{m(\vec{r})}\nabla$, where $m\left(\vec{r}\right)=m\left(r\right)$ and $r=|\vec{r}|\geq 0$. This means that we will investigate the role of a mass which is dependent only on the radial position. This assumption will turn out to be crucial throughout the present work. As we will show, the final results for several mass distributions in 1D behave differently from those in 2D due to the radial symmetry existing in 2D. The ultimate reason has to do mainly with the different boundary conditions at the origin.

## 4. Results for 1D Systems

### 4.1. Ground State

The corresponding stationary Schrödinger’s equation for the 1D system under consideration is given by

(11) $$\psi^{\prime \prime }\left(z\right)-\frac{m^{\prime }\left(z\right)}{m(z)}\;\psi^{\prime }\left(z\right)+2\;E\;m\left(z\right)\psi \left(z\right)=0\;.$$

**Figure 2:**
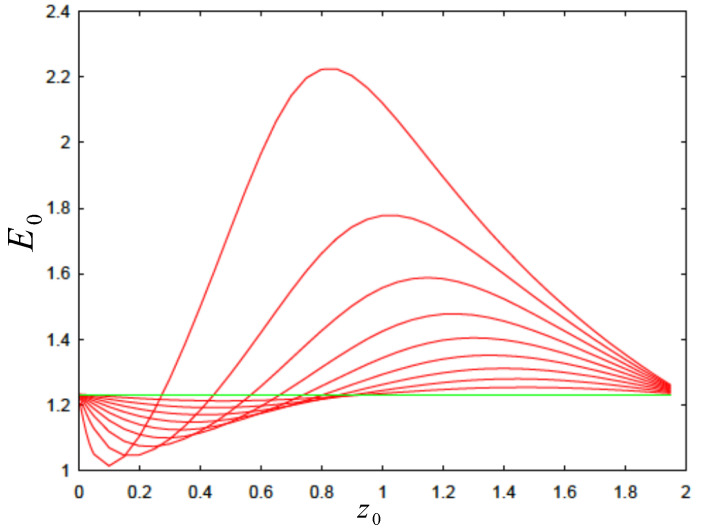
(Color online.) Evolution of the ground state energy, $E_{0}$, as a function of parameter $z_{0}$ for different values of β/α (from top to bottom: 0.1,0.2,…,0.9). The corresponding results belong to the mass distribution in Figure 1a, which is not optimal. However, this structure can be of practical interest. The horizontal line corresponds to $E_{0}=\pi^{2}/8$ for β/α=1. See text for details.

As we shall see, some distributions in Figure 1 are optimal while others are not. When we refer to *optimal* distributions, we imply those piecewise functions for the position-dependent mass, m(z), that retrieves a maximum of energy. For instance, if we consider the case in Figure 1a, which is probably the easiest one to implement experimentally, we obtain some interesting results as depicted in Figure 2. The overall distribution is not globallyoptimal since, depending on the position $z_{0}$, one can have locally maximum or minimum values for the ground state energy $E_{0}$ for any β/α ratio. Although we acknowledge that the distribution is not optimal, it may suffice for all practical purposes.

Let us now consider other distributions in Figure 1 that are optimal in 1D. If the mass is constant near the origin, which is certainly the case for the distributions of m(z) in Figure 1, then we have ψ(0)=0 and $\psi^{\prime }\left(0\right)=1$. The case where the mass is constant returns the usual $\sin(\sqrt{2E}z)$ solution with its known zeroes.

**Figure 3:**
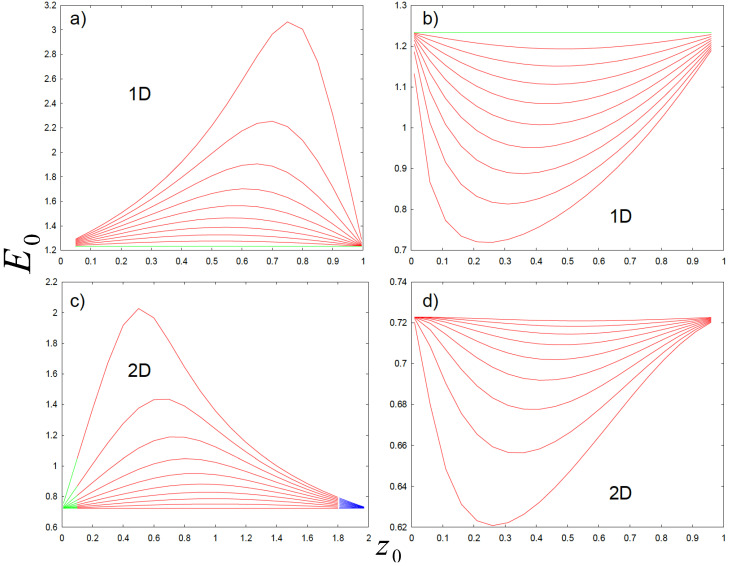
(Color online.) (**a**) depicts maximum energies $E_{0}$ as a function of $z_{0}$ for several values of β/α. (**b**) is equivalent to (**a**) but for minimum values $E_{0}$. (**c**) (with range doubled for the sake of clarity) and (**d**) display the results for the 2D mass distributions being maximum and minimum, respectively. The difference between various mass optimizers in 1D or 2D lies in the diverse concomitant boundary conditions. Horizontal lines correspond to concomitant 1D and 2D values of $E_{0}$ for a constant mass, $m_{0}=1$. Colors have been added at the endpoints in (**c**) to highlight the almost linear behavior at the origin. See text for details.

In Figure 3a, we depict the ensuing ground state $E_{0}$ as a function of the position, $z_{0}$, for several values of β/α. The curves ranging from top to bottom have β/α values of the form 0.1,0.2,…,1. One can easily appreciate the result for α=β. This case coincides with the constant mass, $m_{0}=1$, where $E_{0}=\pi^{2}/8$ (in atomic units as explained earlier). What can be immediately appreciated is that there is indeed a maximal value for $E_{0}$ for each value of β/α and a particular position, $z_{0}$. Therefore, we have indeed found the maximizing distribution m(z) to be of the form of Figure 3a. Remarkably enough, the maximum values $E_{0}^{*}$ and the concomitant values for β/α seem to follow a very interesting functional form that is reminiscent of Wien’s displacement law for black body radiation, $E_{0}^{*}=\frac{\pi^{2}}{8}\;{\left(\beta /\alpha \right)}^{\gamma }$. Actually, this form is quite accurate for γ≈0.39. The functional form for this correspondence is far more complex, but it constitutes a nice and simple approximation to the optimal value $E_{0}^{*}$ with regard to the parameter (β/α).

In Figure 3b, we display the ground state $E_{0}$ as a function of the position, $z_{0}$, for several values of β/α corresponding to the mass distribution in Figure 1c. It is absolutely obvious that mass distributions in Figure 1a,c have very little in common. However, we see that a similar pattern occurs when discussing the results from Figure 3b, but in this case, it is a minimum $E_{0}$.

We can clearly see that a minimum occurs along some value for $z_{0}$ with constant β/α. The curves, again, have (from bottom to top) values of β/α of the form 0.1,0.2,…,1. The situation, α=β, gives rise to the case with constant mass with $E_{0}=\pi^{2}/8$.

When we further analyze the results, we see that the positions $\left(1-z_{0}\right)$ for the minimizer actually match the ones for the maximizer. That is, both the ensuing maximizer and minimizer optimal energies are symmetric with respect to the value of $z_{0}=1/2$. This implies that a common structure must be shared, although it is challenging to know the precise reason.

When considering other expressions for the mass distribution as a function of position, one simple functional form is given by $m\left(z\right)=a\;z^{p}$, where *a* is such that the total mass between 0 and L=2 is the same as in all other distributions. The general solution is physical only in the range between 0≤p≤1, since for p>1, it diverges at the origin. In the case of 0<p≤1, both solutions are of the form $z^{\frac{1-p}{2}}\;J_{\pm \frac{1-p}{p+2}}\left(u\right)$ where the argument, *u* of the Bessel function, is $\frac{2}{p+2}\sqrt{2aE}z^{\frac{p+2}{2}}$. We obtain the final answer by requesting the state to be zero at L=2. However, the eigenvalues become monotonic with m(z) being maximum at p=1. It is certainly intriguing to observe how the convexity of $m\left(z\right)=a\;z^{p}$ plays a major role in the existence of a physical solution.

Very few analytic solutions are available, but among all of them, the most intriguing one is the divergent case of m(z)=a/z. Obviously, this distribution cannot be normalized and, furthermore, it has a singularity at the origin. Nevertheless, it admits an analytic solution of the form $J_{0}\left(2\sqrt{2aE}\sqrt{z}\right)$, which implies $E\propto j_{0,n}^{2}/L$ where $j_{0,n}$ are the zeroes of the Bessel function of the first kind and zeroth order. The fact that the mass is infinite at the origin cannot be explained in terms of the physical meaning of the *actual* mass of the particle. Since we are discussing a position-dependent mass approach for a particle in a quantum well, we have to think about the mass contribution arising as an effective potential term of the form $\propto \hat{\vec{p}}\cdot \nabla m$ after a proper identification of the previous term in the expansion of the second derivative in stationary Schrödinger’s equation.

### 4.2. First Excited State

It is also of some interest to discuss the first excited state (the second eigenvalue) for the 1D case. In order to compare the results, we have solved the same problem as before but for one more energetic level. We have found out that the ratio $E_{1}/E_{0}$ reaches a minimum value exactly at the same position $z_{0}$ where $E_{0}$ is a maximum. Furthermore, the concomitant result increases as α/β increases where values of 0.1,0.2,…,1 are being considered. What is certain is that our computations are in perfect agreement with theory (see M. Ashbaugh and R. Benguria [59]) in the sense of finding the ratio $E_{1}/E_{0}\leq 4$. The equality is attained when α=β which represents the situation when the mass of the particle becomes a constant.

### 4.3. Analytic Investigation

The numerical results mentioned above are supported by some analytical calculations. More precisely, let us consider the 1D case of the problem in Equation (6) given in the following form:

(12) $$\left\{\begin{array}{c} -\frac{d}{dz}\left(\frac{1}{m(z)}\psi^{\prime }\right)=2E\left(m\right)\psi \;\;\mathrm{in}\;\Omega =\left(0,L\right),\\ \psi \left(0\right)=\psi \left(L\right)=0\;. \end{array}\right.$$

Without loss of generality, we keep the constraints used in the above numerical investigations and want to look for a function *m* belonging to the class $F_{\alpha ,\beta ,2}$ that can minimize the ground state energy $E_{0}$. To this end, we follow an idea from S. J. Cox and R. Lipton [63]. We will make use of an appropriate change in variable which allows us to transfer the variable into the lower-order term and, thereafter, use a result from M. G. Krein [64]. More precisely, let us consider any pair ψ and *E* of eigenfunctions and eigenvalues, respectively, for Equation (12) associated to a mass, m(z), belonging to the set $F_{\alpha ,\beta ,2}$. Since m(z)>0 in the interval $\left(0,L\right)$, the function $z⟶\int_{0}^{z}m\left(t\right)dt$ is increasing. Hence, we may consider the following change in variable

(13) $$y=\int_{0}^{z}m\left(t\right)dt\;,$$

and a new function $v\left(y\right)$ given as v(y):=u(z) is introduced. Next, let us denote by $L_{1}$ the maximum value attained by *y*. That is $L_{1}=\int_{0}^{L}m\left(t\right)dt$ and by z(y) the inverse function of $z⟶y\left(t\right)$ defined in $\left[0,L_{1}\right]$. From dy/dz=m(z), we see that *v* satisfies the following equation:

(14) $$\left\{\begin{array}{c} -\frac{d^{2}}{dy^{2}}v=2E\frac{1}{m\left(z\right)}v\left(y\right)\;\mathrm{in}\;\Omega_{1}=\left(0,L_{1}\right),\\ v\left(0\right)=v\left(L_{1}\right)=0\;. \end{array}\right.$$

By introducing a new function

(15) $$\rho \left(y\right):=\frac{1}{m\left(z\left(y\right)\right)}\;,$$

we immediately note that we have 1/β≤ρ(y)≤1/α and $\int_{0}^{L_{1}}\rho \left(y\right)dy=\int_{0}^{L}m\left(z\right)dz=2$ so that $\rho \in F_{1/\beta ,1/\alpha ,2}$. It follows then, due to a theorem of M. G. Krein [64], that

1. The unique minimizer of the ground state $E_{0}\left(m\right)$ in the class $F_{\alpha ,\beta ,2}$ is obtained for the following step-like mass distribution:
  (16) $$m^{*}\left(z\right):=\left\{\begin{array}{c} \beta \;\mathrm{for}\;z\in \left(0,1-\delta \right),\\ \alpha \;\mathrm{for}\;z\in \left(1-\delta ,1+\delta \right),\\ \beta \;\mathrm{for}\;z\in \left(1+\delta ,2\right), \end{array}\right.$$
  where δ=α(β−1)/(β−α).
2. The unique maximizer of the ground state $E_{0}\left(m\right)$ in the class $F_{\alpha ,\beta ,2}$ is obtained for the following step-like mass distribution:
  (17) $$m^{*}\left(z\right):=\left\{\begin{array}{c} \alpha \;\mathrm{for}\;z\in \left(0,1-\delta \right),\\ \beta \;\mathrm{for}\;z\in \left(1-\delta ,1+\delta \right),\\ \alpha \;\mathrm{for}\;z\in \left(1+\delta ,2\right)\;, \end{array}\right.$$
  where δ=β(1−α)/(β−α).

Finally, we note that Krein’s theorem addresses the extreme values of each eigenvalue of Equation (14) under the assumption that the function ρ, defined in Equation (15), is bounded uniformly from below and above and has a fixed mean. Therefore, Krein’s theorem may also be used here to obtain similar results for higher-order eigenvalues of Equation (12) where the optimal values of $E_{k}$ are obtained for some periodic step-like mass distributions (see M. G. Krein [64] for more details).

## 5. Results for 2D Systems

Realistic 2D systems of electrons can be created in semiconductor heterojunctions and/or heterostructures. A commonly studied heterojunction is one that combines GaAs and AlGaAs (GaAs dopped with Al). Both GaAs and AlGaAs have the same crystal structure, but AlGaAs has a wider band gap than GaAs. As a result, electrons originally on the AlGaAs side of the interface can lower their energy by moving across the interface to the GaAs side. The net result is the creation of an almost perfect 2D system of electrons at the interface [65,66]. For this particular example (GaAs/AlGaAs heterojunction), the effective mass of the electrons is constant (though much smaller than the bare electron’s mass). However, one can envision more complicated scenarios in which the electrons have a more complex position-dependent mass. A 2D setup that is challenging but still amenable to an analytical treatment is that of a particle that has a position-dependent mass which is a function solely of the radial variable, as follows:

(18) $$m\left(\vec{r}\right)=m\left(r\right)\;,$$

where $r=|\vec{r}|=\sqrt{x^{2}+y^{2}}$ in 2D. The corresponding stationary Schrödinger’s equation for the radial part R(r) of the wave function (the angular part just gives rise to the usual circular functions imposing periodicity) is given by

(19) $$r^{2}R^{\prime \prime }\left(r\right)+\left[r-r^{2}\frac{m^{\prime }\left(r\right)}{m(r)}\right]R^{\prime }\left(r\right)+\left[2\;E\;m\left(r\right)\;r^{2}-n^{2}\right]R\left(r\right)=0\;,$$

where n=0,1,2,… are the radial quantum numbers. If the mass is constant near the origin, as it is the case for mass distributions in Figure 1, then we have R(0)=1 and $R^{\prime }\left(0\right)=0$. The previous equation leads to the usual Bessel functions of the first kind, $J_{n}\left(\sqrt{2E}r\right)$ for $m\left(r\right)=m_{0}$. One notes that the 1D radial differential equation for R(r) in Equation (19) was obtained only because of the assumption of radial symmetry for the position-dependent mass.

The results in the 2D case scenario are different from those for the 1D counterpart. We see that $E_{0}$ depicted in Figure 3a is different from the one depicted in Figure 3c. The concomitant result is definitely a maximum. The surprising thing is that it happens for different step-wise mass distributions as Figure 1a in this case.

However, the minimum case scenario in Figure 3d coincides with the one in 1D. The 2D case with mass that has radial symmetry has a completely different differential equation, yet the mathematical problem remains the same, except for the boundary conditions. There is no mathematical theorem that defines the optimal shape for the position-dependent mass, m(r). However, what we can say is that its existence and uniqueness are warranted. The message from Figure 3 is that there are two mass distributions that are most likely to be the ones.

As in the 1D case, a position-dependent mass such as m(r)=a/r allows for an analytic result. The solution of the radial differential equations turns out to be of the form $J_{1}\left(u\right)$ with argument $u=2\sqrt{2aEr}$ which is convergent everywhere. The discussion of the validity of the usual approach for particles with position-dependent mass remains open, unless some effective potential analogy is made.

## 6. Conclusions

We discussed in detail the mathematical implications of having a particle with position-dependent mass m(z) as an optimizer for $E_{0}$ and $E_{1}$ in 1D. We also considered a 2D quantum well scenario where we assumed a radially dependent mass of the form $m\left(\vec{r}\right)=m\left(r\right)$, where $r=|\vec{r}|\geq 0$ is the 2D radial variable. In both cases, the kinetic energy term in Schrödinger’s equation is of the form $-\frac{1}{2}\nabla \frac{1}{m(\vec{r})}\nabla$ with the proper interpretation of the gradient operator in terms of the dimensionality of space. We obtained analytical and numerical results supporting specific step-like mass distributions which are not difficult to implement experimentally. Overall, our conclusions agree with the analysis of the results found. Therefore, it is expected that our contribution will shed some light on phenomena involving semiconductor materials, for instance, in designing new semiconductor nanodevices with properties tailored to meet specific requirements.

Obviously, questions can be asked about the present approach for those mass distributions that give rise to analytic solutions, whereas the functional form for the position-dependent mass is clearly divergent. In other words, having a solution that is mathematically regular at a pole of m(z) is physically questionable, especially if m(z) represents an effective form for the mass. Of course, the effective mass is related to the band structure, but in any case, singularities always tend to be cumbersome. Therefore, this fact by no means can be acceptable from a physical point of view, unless some constraints are imposed within the position-dependent mass approach employed in the present work.

A few other questions that may rise from the approach carried out in the present work are listed below:

(i) The spatial dependence of the effective mass of a particle in a given realistic semiconductor heterostructure is caused by the spatial dependence of the composition of the material (e.g., the content of Al in a GaAs/AlGaAs heterojunction). Crucially, this spatial variation in composition results in spatial dependence of all the other band-structure parameters and not only the effective mass. In the minimal single-band model considered here, it means that the energy of the minimum of the conduction band (assuming for concreteness that we are talking about electrons in the conduction band) should be spatially dependent. This would imply that for any realistic semiconductor heterostructure, there should be an extra term (written in 1D notation) in the left-hand side of Equation (6) of the form $2\;E_{m(z)}\;\psi \left(z\right)$ with $E_{m(z)}$ being the energy of band minimum at a given position.
(ii) The addition of the previous term in the problem of mass distribution optimization would provide a natural constraint for the optimization problem. This would also remove the need for the introduction of the constraint $\int_{\Omega }m\left(z\right)\;dz=c$, which is essential in the connection made between the quantum problem and the maximum principle in the theory of elliptic differential equations.
(iii) The envelope function ψ(z) is not exactly zero at the boundaries. However, in most cases, the transport occurs in other directions. For example, in most transport experiments involving quantum wells, the current flows in the plane of the quantum well. Namely, charge carriers are free to move in the *x*-*y* plane under the assumption that the quantum well confinement is in the *z*-direction. However, a more careful treatment of wave functions at well boundaries is of course needed when tunneling in a direction perpendicular to the plane of a quantum well (in the *z*-direction).
(iv) An infinite effective mass means that the band is flat. This can happen (approximately) for realistic band structures. However, based on our knowledge, it does not happen for alloys of popular semiconductors that are used to manufacture heterostructures.

At this juncture, we take the opportunity to remark that, in our view, the contribution spelled out in this work represents a brand new approach (perhaps a zero-th order approach) that connects the quantum problem of a particle with position-dependent mass with some well-known results in the theory of elliptic differential equations. This means that it is possible to relate possible experimental applications in the field of low-dimensional semiconductors with purely mathematical results as those concerning the maximum principle in the theory of elliptic equations. It would have been very beneficial to compare the present results with corresponding available results in the literature obtained by other methods. However, we are not aware of the existence of other cases in the literature where such a comparison is feasible. To the best of our knowledge, little is found in the literature for the main case of our study, that is, a particle with piecewise constant effective mass in 1D or 2D. Nevertheless, we are confident on the accuracy of the present numerical results which, in our opinion, can be easily reproduced by other researchers if need arises. As a final note, we point out that in our study, there is no external potential considered either in the 1D, or in the 2D case, except for the presence of the infinite walls at the boundary. This means that the properties of the system under consideration are solely determined by the piecewise constant effective mass of the electron together with the boundary conditions. This is unlike some prior studies that we are aware of, for instance, studies of 1D classical and quantum oscillators with position-dependent mass [57].

## Data Availability

The data presented in this study are available upon request from the authors.
